# A path toward disability-inclusive health in Zimbabwe Part 2: A qualitative study on the national response to COVID-19

**DOI:** 10.4102/ajod.v11i0.991

**Published:** 2022-05-30

**Authors:** Tracey Smythe, Thubelihle Mabhena, Shepherd Murahwi, Tapiwanashe Kujinga, Hannah Kuper, Simbarashe Rusakaniko

**Affiliations:** 1 Department of Clinical Research, Faculty of Infectious and Tropical Diseases, London School of Hygiene and Tropical Medicine, London, United Kingdom; 2 Pan African Treatment Access Movement, Harare, Zimbabwe; 3 Leonard Cheshire Disability Zimbabwe, Harare, Zimbabwe; 4 Department of Community Medicine, University of Zimbabwe, Harare, Zimbabwe

**Keywords:** disability, COVID-19, Zimbabwe, qualitative, equity, Missing Billion, inclusion, health system, health access

## Abstract

**Background:**

People with disabilities are at higher risk of adverse coronavirus disease 2019 (COVID-19) outcomes. Additionally, measures to mitigate COVID-19 transmission have impacted health service provision and access, which may particularly disadvantage people with disabilities.

**Objectives:**

To explore the perspectives and experiences of people with disabilities in accessing health services in Zimbabwe during the pandemic, to identify perceived challenges and facilitators to inclusive health and key actions to improve accessibility.

**Methods:**

We used in-depth interviews with 24 people with disabilities (identified through purposive sampling) and with 10 key informants (from expert recommendation) to explore the impact of COVID-19 on access to health care. Interviews were transcribed, coded and thematically analysed. We used the disability-inclusive health ‘Missing Billion’ framework to map and inform barriers to inclusive health care during COVID-19 and disparities in outcomes faced by people with disabilities.

**Results:**

People with disabilities demonstrated good awareness of COVID-19 mitigation strategies, but faced difficulties accessing COVID-19 information and health services. Challenges to the implementation of COVID-19 guidelines related to a person’s functional impairment and financial ability to do so. A key supply-side constraint was the perceived de-prioritisation of rehabilitation services. Further restrictions on access to health services and rehabilitation decreased an individual’s functional ability and exacerbated pre-existing conditions.

**Conclusion:**

The immediate health and financial impacts of the COVID-19 pandemic on people with disabilities in Zimbabwe were severe. Government departments should include people with disabilities in all communications and activities related to the pandemic through a twin-track approach, meaning inclusion in mainstream activities and targeting with specific interventions where necessary.

## Background

People with disabilities experience inequities in health access and outcomes, and these are potentially magnified with respect to coronavirus disease 2019 (COVID-19) (Shakespeare, Ndagire & Seketi 2021a). Estimates from the UK show that at least 58% of deaths related to COVID-19 between January 2020 and February 2021 were amongst people with disabilities, although they only made up 17% of the population (Bosworth et al. 2021). The increased risk of people with disabilities to COVID-19 mortality appears to occur for a combination of reasons, including higher risk of contracting the disease (e.g. difficulties socially isolating, lack of accessible guidance on preventing infection) and vulnerability to more severe morbidity (e.g. on average older with more pre-existing conditions and more likely to live in deprived circumstances) (Shakespeare et al. 2021). People with disabilities will also often need routine health care, such as a supply of medication and physiotherapy, which may be disrupted because of COVID-19, leading to further decreased physical and mental health and functioning (Shakespeare et al. 2021b; Steptoe & Di Gessa 2021).

Limited available data suggests that the impacts of COVID-19 on people with disabilities are also noted in low and middle-income countries (LMICs), including Zimbabwe. For instance, a rapid mixed-methods review on the impacts of COVID-19 on people with disabilities and its effect on the health delivery system in Zimbabwe found that there were no structures in place in 2020 to accommodate testing of people with disabilities. They had limited access to COVID-19 information, and most health delivery services were inaccessible (Manikai 2020). Organisations of persons with disabilities (OPDs) were not actively engaged or consulted in the formulation, development or implementation of the National COVID-19 Response Plan (Manikai 2020). The report concludes that the barriers to accessing routine health care and the inequity that people with disabilities experience has limited their self-efficacy and further marginalised people with disabilities in Zimbabwe. Moreover, health workers found implementing the measures particularly difficult without access to water, personal protective equipment (PPE) and daily income (Mackworth-Young et al. 2021). The devastating economic effect that public health measures such as curfews, bans on transport and lockdowns have on populations that are largely dependent on the informal economic sector were also highlighted (Dzobo, Chitungo & Dzinamarira 2020).

**FIGURE 1:**
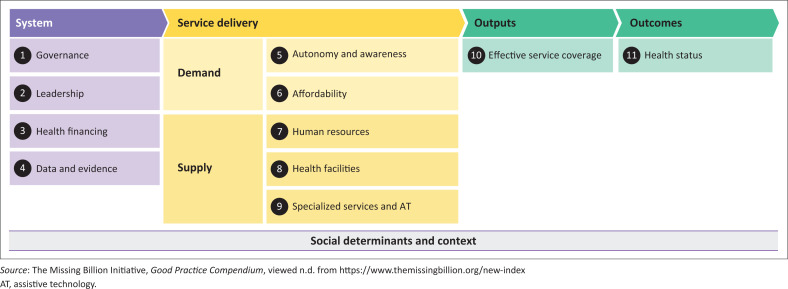
Preliminary framework of inclusive health systems.

There has been a lack of consideration, however, of the experiences of people with disabilities in accessing health care during COVID-19 and the impact thereof on health outcomes in Zimbabwe, or other LMICs. This information is needed to inform how health systems should be strengthened to offer a disability-inclusive COVID-19 response. The ‘Missing Billion’ Report (Hogan 2020; Litullo 2019) (Figure 1) provides a framework for how to identify components of the health system that require strengthening in order to provide disability-inclusive health. The framework proposes consideration of barriers and facilitators from the perspective of people with disabilities – ‘demand’ (e.g. affordability), service providers – ‘supply’ (e.g. accessible health facilities) and at the systems level (e.g. leadership).

Evidence is lacking for Zimbabwe on what the perceived challenges and facilitators were to inclusive health during the COVID-19 pandemic, and key actions to improve accessibility and design of the health system for inclusion. We aimed to explore the perspectives and experiences of people with disabilities in accessing health services in Zimbabwe during the pandemic to identify perceived challenges and facilitators to inclusive health and key actions to improve accessibility.

## Methods

Our methodological approach and consequent reporting were underpinned by the Consolidated Criteria for Reporting Qualitative Research (COREQ) statement, which is a 32-item checklist (Tong, Sainsbury & Craig 2007).

### Study design

This qualitative study was undertaken alongside a companion study to understand the access to health services for people with disabilities in Zimbabwe prior to COVID-19 (Smythe et al. in press). This qualitative study focused on experiences and perceptions of people with disabilities in accessing health care during the COVID-19 pandemic and key actions to improve accessibility.

### Setting

The study was conducted in the capital city Harare and rural and urban areas of Gutu between 31 May and 12 June 2021. The focus was on the public health sector that primarily services the low-income and informal settlement populations. Participants included 24 people with disabilities in Gutu and 10 key informants from local and national health authorities in Gutu and Harare.

### Participants

We used purposive sampling to recruit people with disabilities through non-governmental organisations (NGOs) and OPDs. The NGOs and OPDs recommended information-rich cases and provided the researchers with a contact list. People with disabilities were then purposively selected to ensure representation by impairment type or condition (e.g. physical, sensory, intellectual), age (children, working-age, older adults), gender and level of support needed for daily life (e.g. none or minimal, ongoing health care or social service needs, requiring carer support for activities of daily living) (Table 1). We used expert recommendation to recruit key informants. Key informants were selected based on their pivotal role and experience in disability programming. All participants were approached through telephone calls. Two individuals who agreed to be interviewed were subsequently unable to take part in the interviews because of health-related concerns.

**TABLE 1:** Demographics of people with disabilities by impairment category†, age and sex.

Category	Frequency (*n*)
**Impairment/condition** †	
Epilepsy	4
Intellectual/behavioural	4
Physical impairment	8
Speech and/or hearing impairment	5
Visual impairment	5
**Age (years)**	
< 10	2
10–20	3
21–30	5
31–40	3
41–50	2
50+	9
**Sex**	
Female	13
Male	11

† , 2 participants had more than one impairment

Data were collected through in-person interviews at the home of people with disabilities and at the place of work of key informants. The majority of participants were interviewed face-to-face. Carer or proxy interviews were used for children below the age of consent (10 years as per national guidelines) and for people with severe difficulties understanding or communicating even with available adaptations (e.g. people with hearing loss, illiterate and with no knowledge of sign language; people with severe intellectual or cognitive impairments). Children aged 10 years or older but below the age of consent participated in interviews with parental consent and individual assent.

Inclusion of people with disabilities was supported through the provision of psychological support services when needed, sign language interpretation, accessible interview sites and transport, use of available district psychological services and researchers skilled at communicating with people with cognitive impairments.

### Data collection

Interview guides with questions and prompts (Appendices 1 and 2) were developed and cognitively tested for understanding and administered in English or Shona by trained research assistants. The research assistants were three women with disabilities who had completed tertiary education. They underwent a one-day online training that included presentation of the study protocol and qualitative methods. Next, they attended a two-day in-person training on data collection, with ongoing mentoring and support provided by the study team. No interviews were repeated and transcripts were not returned to participants for comment. Interviews took between 30 and 60 min and were audio recorded with written consent from the participants. Field notes were made during and after the interviews.

### Data management and analysis

These processes were the same as for the companion paper (Smythe et al., in press). All interviews were transcribed verbatim for analysis and translated into English where necessary. Data were managed using NVivo 12. Interview transcripts and detailed notes were analysed using thematic analysis (Guest, Macqueen & Namey 2012). A coding framework was developed using the semi-structured interview guide as a starting point, which was adapted to include additional codes and themes emerging from the data. An allied health professional and epidemiologist from Zimbabwe, with experience in both qualitative and quantitative research methods (TS) coded the interview transcripts to identify the key themes emerging from the data. These were discussed across the entire team, including the research assistants, and analysis was evaluated by research team members (TM, SM, TK and SR), to ensure that interpretations were credible and valid. Regular discussions with the research team took place throughout the data analysis phase to ensure content validity and context.

To explore inclusive health practices during the COVID-19 pandemic, we applied the Missing Billion health system framework (Figure 1) (Hogan 2020; Litullo 2019) to map and inform the perceived and experienced barriers and facilitators to health services. We undertook a narrative synthesis of the findings and reported the results alongside the framework. The predominant focus was on the service delivery components of the framework, including the demand and supply-side perspectives.

### Ethical considerations

Ethical approval for the study was granted from the Medical Research Council of Zimbabwe (MRCZ) (No MRCZ/A/2731) and the Institutional Review Board at London School of Hygiene & Topical Medicine (No 22138 – 2).

The main ethical considerations were the same as for the companion study (Authors, under review, Part 1). We managed participant expectations by describing in detail the nature and detail of our study. We assured confidentiality by not linking any data to particular participants. Informed consent was sought after providing a written information sheet and reiterating the information verbally in the language of choice (English or Shona). The research assistants facilitated referrals, as necessary, to medical services and/or OPDs. All interviewees were compensated for their time and transport was reimbursed.

## Results

Data are presented under the five themes that comprise the Missing Billion framework (Kuper & Heydt 2019) on demand and supply side factors for service delivery: demand – autonomy and awareness, affordability; supply – human resources, health facilities, specialised services and assistive technology. Table 2 provides an overview of the themes and sub-themes identified.

**TABLE 2:** Overview of themes.

Themes	Sub-themes
**Demand**	
Autonomy and awareness	Health literacy related to COVID-19 Health beliefs Functional ability to adhere to guidance Access to transport Perceived excess risk of COVID-19
Affordability	Out-of-pocket payments Transport costs Opportunity costs in seeking care Access to social protection
**Supply**	
Human resources and health facilities	Supply chain disruptions Healthcare provider knowledge, attitude and competence Community support Outreach services
Specialised services and Assistive Technology	De-prioritisation of rehabilitation services Ability to engage Satisfaction with care

### Demand-side factors

Challenges and facilitators from the perspective and experience of the person with disabilities (i.e. ‘demand-side’) were observed in awareness about COVID-19 mitigation, autonomy to implement these strategies, and affordability.

### Demand – Autonomy and awareness

People with disabilities generally demonstrated good awareness of COVID-19 mitigation strategies, and required actions. Masking, washing hands and keeping distance from others were most commonly mentioned. These measures were mainly learnt about through the radio, and from community leaders. Caregivers spoke of people with hearing impairments facing problems with communication; they were excluded from understanding messaging on the radio, which left them marginalised from the spoken world. In addition, masking created a barrier to communication, where:

‘There is need for lip reading but unfortunately, because of the mask, she cannot read the lips.’ (Participant 23)

People with visual impairment also reported informational barriers as they were reliant on others to share current information:

‘I cannot see like I used to. It’s frustrating for me because when an emergency message comes on my phone, I can’t read it and I have to wait for someone to return home.’ (Participant 21)

One of the OPDs that we interviewed highlighted that people with disabilities had many unanswered questions and did not often know what information to believe. The rumours spread fear and uncertainty, for example:

‘There was fear among them because of the things that were being said about COVID-19 and the regulations were imposed on top of that. There are the rumours that COVID-19 survives on metallic surfaces for long period of time. People use walking sticks and white canes, and they continuously touch and hold for them for long periods of time. There is a great need for health officials to come and educate us on the actual facts in relation to COVID-19.’ (Key Informant 03)

Difficulties arose for people with disabilities in the implementation of COVID-19 guidelines. Many caregivers and people with disabilities experienced exclusion, fear and pain when attempting to follow national guidelines. The demands of physical distancing limited people’s ability to communicate and participate in daily life. People with physical impairment who required a caregiver for mobility experienced greater isolation and limited independence because of caregivers’ fear of being unable to comply with physical distancing guidelines:

‘Washing hands after every contact is impossible. Social distancing is another challenge. If I’m in a wheelchair and social distance is not possible, then what will law enforcers do to me because I’m not following regulations? This is why most disabled people are not moving, because the ones who are supposed to escort them won’t show up.’ (Key Informant 03)

Attempting to adhere to recommendations created pain and discomfort for people with albinism, who have skin that is sensitive:

‘The sanitisers that I have used have burnt my skin. It usually stings when I use sanitiser.’ (Participant 22)

With regard to the ability to adhere to guidance, the main challenges to implementation of COVID-19 guidelines related to a person’s functional impairment and financial ability to do so. Physical distancing and self-isolation measures were not feasible for some people with disabilities who relied on caregivers. Those that required assistance for daily activities experienced greater anxiety with regard to physical contact with their caregiver, and in relation to fear of transmission of COVID-19 through their metal assistive product. For example:

‘I’m very concerned about COVID-19. I am always in contact with people who assist me on a daily basis. Sometimes those people are not putting on face masks. From what I heard on the radio, people are supposed to sit some distance from each other and not hold hands.’ (Participant 17)

People with physical impairments who required assistance for mobility experienced limited ability to access water, and people with visual impairments voiced concern about whether the water would be clean or not:

‘Whilst I have the knowledge, I need help to implement the guidelines with someone who is careful because I cannot see.’ (Participant 06)

Caregivers reported that explaining mitigation strategies to people with intellectual impairment was challenging and often unsuccessful:

‘If I give her a mask to wear, she usually tears it off.’ (Participant 13)

‘You talk of persons with intellectual disabilities who are not able to interpret simple instructions like telling them to wear a mask. It doesn’t make sense to them; why should they be wearing a mask and why wear it daily?’ (Key Informant 10)

The lack of access to transport to attend hospital check-ups and the presence of roadblocks to minimise movement led to additional challenges for people with disabilities and impacted their functioning. While the restrictions were the same for everyone, the ability to follow these measures was further limited by administration requirements to access transport. This difficulty was also reported by many OPDs. One representative highlighted:

‘It was difficult for our clients to get authorisation to travel, either to travel to access health services at health centres … they had to go to the headman or chief to get a letter.’ (Key Informant 10)

Regarding the perceived excess risks of COVID-19, people with disabilities viewed the risk of contracting COVID-19 differently, and this perception depended on their social contact, beliefs about their physical strength, the state of their immune systems, and the extent to which they were reliant on others for daily care. Those who were already isolated did not perceive COVID-19 as a greater risk than people without disabilities, and they reported spending most of the day alone and with no meaningful activity and contact with other individuals:

‘My chances of getting it are reduced because I do not usually meet up with people. I don’t often leave my home so I do not meet a lot of people.’ (Participant 15)

‘It [the excess risk] is not high because most of the time I’m alone at home.’ (Participant 21)

The pandemic highlighted the extent of how fragile the social bonds for people with disabilities were, and a key feature was perceived personal vulnerability because of being reliant on others. Participants felt an extra burden of responsibility to protect themselves and their own health, whilst the perception was that people without disabilities may not adhere to COVID-19 regulations as required:

‘I am at the mercy of other people. Maybe what they may do to me is not what is required during this period of this disease.’ (Participant 19)

However, vulnerability was felt more acutely by those who perceived their bodies to be weaker:

‘This disease has troubled us, especially us, who have disabilities and other diseases. All I can say is that it is time for the survival of the fittest.’ (Participant 18)

Anxiety and strain were also experienced by caregivers who perceived people with disabilities as being at greater risk of contracting COVID-19, as summarised by the following quotes:

‘He does not know that he has to wear a mask. He may become exposed; that is where my concern is.’ (Participant 01)

‘I do not feel comfortable leaving him with his peers. He does not communicate well and this might increase his risk in contracting Covid.’ (Participant 14)

‘He is at a high risk because there is not much he can do without being assisted by me or anyone else … he needs help to eat, to wash his hands or to be generally more comfortable.’ (Participant 16)

‘She does not know the signs, and if she gets to a place with many people who cannot communicate with her to warn her, she might get infected.’ (Participant 20)

This meant that additional strain was experienced by caregivers as they did not rely on external help. The perceived need to keep people with disabilities safe created pressure for families.

### Demand – Affordability

Lack of affordability created tensions between public health advice and the ability to practise them effectively:

‘I have to take the money for a mask from my budget, and sometimes there won’t be any sugar or salt in the house.’ (Participant 02)

When asked what is used when there is no soap, one person with disability replied that where water was scarce or inaccessible that ‘we use ashes’ (Participant 23).

People with disabilities felt forgotten and alone when faced with the additional challenge of having to prioritise their general health and prevent further impairment against that of COVID-19 prevention measures:

‘There is no money, my medication for my eyes needs US$3 at the pharmacy, there is no way I will ask for a sanitiser whilst I have problems with my eyes. I would rather be fighting for my eyesight so that I will not be blind forever.’ (Participant 21)

Increased pressures on social protection schemes intensified economic and social exclusion of persons with disabilities. People with disabilities believed that their livelihoods were disproportionately impacted and this was reinforced by the experiences of OPDs:

‘Before the pandemic there were people who needed food, but the number has increased because of the pandemic…it means the competition is stiffer.’ (Key Informant 05)

Livelihoods were linked to quality of, and ability to access health services. Whilst some health services were offered for free at clinics, NGOs and representatives for mission hospitals no longer came to local clinics, and if medications or services were not available, people with disabilities had no other option because of their limited ability to pay. People with disabilities experienced a disproportional impact and consequences of the already inadequate health systems.

### Supply-side factors

Challenges and facilitators from the perspective of the health system (i.e. ‘supply-side’) were observed in human resources, appropriate health facilities and specialised services.

### Supply – Health facility availability

Measures to mitigate COVID-19 transmission have directly and indirectly impacted health service provision and access, including through supply chain disruption and diverting resources. People with disabilities believed that their needs did not matter as restrictions mandated by the government, which were aimed at stemming the spread of COVID-19, severely limited their access to basic health services:

‘During COVID it was difficult to go for a check-up … they only wanted people who were seriously ill.’ (Participant 22)

Health care provider attitude and competence emerged as factors influencing the perceived supply of health services. People with disabilities avoided seeking health services due not only to fears of becoming infected with COVID-19 and the punitive action for breaching measures such as movement restrictions, but also as a result of the poor interpersonal relationship with health care providers:

‘We were scared but travelling during that time was even scarier. Other people informed us that it was pointless to go to the hospital because nurses were said to be reluctant to serve people and consulted people from a distance.’ (Participant 17)

Greater disruptions in other medical supply chains further limited health service provision:

‘The hospital is also struggling to get transport to collect certain important resources.’ (Key Informant 05)

Supply of medications were limited at source, and OPDs no longer received their regular donations:

‘Before the COVID pandemic started we used to get a variety of stuff, including sunscreen, from different organisations … but when COVID started, all that aid stopped being availed.’ (Key Informant 01)

Different mitigation strategies were implemented to overcome these gaps in availability. For instance, community support and ‘togetherness’ was key to being able to access basic health needs:

‘Since most of us could not manage to go to Gutu, our disability group leader suggested that we contribute money and send one person there to collect pills for everyone. Then the pills would be distributed among us when that person returned.’ (Participant 15)

There were some examples where the health services helped overcome these issues by strengthening outreach to the community:

‘Our mobile clinic and our general model of operation ensured that people had ART medication throughout the pandemic and HIV testing was available to whosoever desired it without interruption during the COVID era.’ (Key Informant 04)

### Supply – Specialised services and assistive technology

Rehabilitation services were deprioritised because of the COVID-19 exposure risk to patients and staff: ‘Rehabilitation centres were not available because they involve a lot of physical contact, so the government dissuaded people from running operations during the pandemic’ (Key Informant 10). However, the services remain limited as ‘community rehabilitation is not being practised these days because of lack of resources’ (Key Informant 05). The need for assistive products and interventions that can optimise functioning do not stop because of COVID-19, yet services to provide these have been reduced:

‘When COVID started, things changed; I stopped going for physiotherapy.’ (Participant 25)

This reinforced the belief of people with disabilities that their lives are less valued in Zimbabwe society, as demonstrated by the following quotes: ‘After being looked down upon, this type of treatment also reduces the confidence of disabled people’ (Key Informant 01), and ‘we feel like the government is not interested in addressing the needs of people with disabilities. NGOs come here and they don’t include us either’ (Key Informant 03).

### Outcomes and impact on functioning

All participants raised concerns that a singular focus on prevention and treatment of COVID-19 led to a severe disruption in medical treatment, health services and rehabilitation. Long periods of isolation heightened mental health, economic and financial pressures, and all people with disabilities that we spoke to shared a belief that they experienced worsening health and well-being during this time:

‘When the seizures come again because of not taking pills, the seizures are more powerful. There are times when I would spend more than a week not knowing where I was or what I was doing, or times when I went for two or three days without eating because of powerful seizures.’ (Participant 15)

People with disabilities reported experiencing burns from uncontrolled seizures near open fires, worsening eyesight without access to glaucoma medication (via eye drops that reduce eye pressure and thereby protecting the optic nerve) and poorer mental health as a result of psychiatric medications being unavailable at clinics and unaffordable at private chemists.

The well-being of people with disabilities was also affected, and they reported increased fear and anxiety that centred around medical stockouts and deterioration of their pre-existing condition:

‘When I don’t have pills, I fear going to the garden or doing other tasks by myself. An epileptic episode can occur anytime, and I may collapse. Fetching water or cooking on fire is daunting for me.’ (Participant 12)

The national COVID-19 response limited access to both general health care and rehabilitation, which exacerbated pre-existing conditions and decreased the functional abilities of people with disabilities.

## Discussion

The immediate health and financial impacts of the COVID-19 pandemic on people with disabilities have been disproportionate and severe. Our study has highlighted inequities in society and structural shortcomings within Zimbabwe where the needs of people with disabilities have not been protected in rural or urban areas. While people with disabilities demonstrated good awareness of COVID-19 mitigation strategies, demand was limited by difficulty accessing COVID-19 information and health services. A person’s functional impairment and socio-economic status were barriers to implementation of COVID-19 guidelines. Supply was constrained by perceived de-prioritisation of rehabilitation services; people with disabilities felt abandoned and forgotten and experienced heightened fear and anxiety. Further restrictions on access to health services and rehabilitation decreased their functional ability and exacerbated pre-existing conditions. Uncertainty in outcome or standard of care emerged as a key recurring experience, which created distress, a sense of fear and a loss of hope.

Further deprioritising people with disabilities during the COVID-19 pandemic has heightened their marginalisation and experiences of inequity. Barriers to accessing care were similar to non-pandemic times (Authors, under review, Part 1), where the demand for health services was limited by health literacy affordability of services, and supply of health services was constrained by perceived poor capacity of health workers to treat people with disabilities, discrimination and inaccessible information and infrastructure. This study found that these everyday barriers were heightened during the pandemic, for example, where physical accessibility affected implementing basic hygiene measures. Our data call attention to the exclusion of people with disabilities and suggest that many people with disabilities and their families have felt abandoned and forgotten during the pandemic, similar to other findings globally (Shakespeare et al. 2021). People with disabilities have not been considered or involved in planning of measures taken to contain the COVID-19 pandemic. A qualitative analysis of media from Ghana, Guinea, Liberia, Niger, Nigeria and Sierra Leone found that people with disabilities were often not consulted during policymaking and thus were deprived from effectively benefitting from the special initiatives that governments took to fight COVID-19 (Saalim et al. 2021). This is a pattern that has been repeated (Kuper et al. 2020; Reichenberger et al. 2020; Shakespeare et al. 2021a, 2021b).

Our key findings are similar to those in South Africa, where exclusion of people with disabilities was exacerbated by the national COVID-19 response (Ned et al. 2020). Key disability-specific health services were not considered as essential services during the initial stages of lockdown, and people with disabilities experienced limited healthcare and rehabilitation access, which was influenced by structural failings (McKinney, McKinney & Swartz 2021). A qualitative study in Uganda found that the national COVID-19 response limited access to health and rehabilitation services for children with disabilities and called for greater attention to this marginalised group and their families when implementing mitigation measures and long-term responses (Mbazzi et al. 2021).

The need for additional support and targeted mental health services was highlighted by the impact of COVID-19 on anxiety and depression. During the first COVID-19 lockdown in Zambia and Sierra Leone, a survey of 468 children and young people who have disabilities and are disadvantaged found that participants had increased anxiety and fear (Sharpe et al. 2021). In Ethiopia, a high prevalence of depression, anxiety and insomnia was found in 423 respondents of a survey of people with disabilities (Necho et al. 2021) and these findings are echoed in the UK, where people with physical disabilities were found to be at particular risk for emotional distress, poor quality of life and low wellbeing during the COVID-19 pandemic (Steptoe & Di Gessa 2021).

With regard to meeting daily needs, the survey in Zambia and Sierra Leone (Sharpe et al. 2021) also showed that nearly 91% of participants reported that they needed considerable additional support with regard to finance, food and schooling. People with disabilities in the UK also experienced difficulty in meeting their daily needs (Shakespeare et al. 2021b). In Uganda, families of children with disabilities had difficulties meeting daily basic needs as they were unable to work and had no income during the COVID-19 related lockdown (Mbazzi et al. 2021).

Despite these findings on impact on healthcare and rehabilitation access, mental health and meeting everyday needs, there remain gaps in evidence. While there is evidence for impact on mortality for the UK, where 58% of deaths related to COVID-19 between January 2020 and February 2021 were amongst people with disabilities, although they only made up 17% of the population (Bosworth et al. 2021), data are limited in other settings. There is a lack of data being collected at national and international levels on impacts on people with disabilities, both in terms of infection and mortality rates, and the impact on poverty, employment, education and isolation in the community (Meaney-Davis 2020).

Our study has limitations that need to be considered when interpreting the results. This is a qualitative study, limited to a modest-sized group of people with disabilities living in rural and urban areas of Zimbabwe. A larger sample could have improved inter-group comparisons (e.g. differences between age groups) and generalisability to different health system contexts. Nevertheless, checks were in place to strengthen the integrity of data and interpretations, which included researchers with disabilities being trained to undertake the qualitative data collection, which likely improved data quality through strengthening the rapport of the interviewer and participant. However, the interviewers may also have brought their own biases to the interview, based on their personal experiences. Additionally, no interviews were repeated nor transcripts returned to participants for comment, and all the transcripts were coded by a single coder. We therefore had several checks in place to strengthen the integrity of data and interpretations. These included involving research assistants, who collected the interviews in data analysis and interpretation, and ongoing discussions amongst the whole team throughout data collection and analysis, particularly on our positionality and reflexivity. The Missing Billion framework provided a structure for consideration of challenges and solutions to inclusive health. We used this framework to consider demand and supply-side service delivery factors in this study but did not address systems-level factors such as governance and leadership. Strengths of our study include that face-to-face interviews were possible within the timeframe of the national COVID-19 response. We achieved both breadth and depth of functional impairment and age range.

There are many important lessons that are being learned on how to create a disability-inclusive COVID-19 response, including in low-resources settings. Government departments should meaningfully engage people with disabilities or their representative organisations to facilitate appropriate planning. A twin-track approach that addresses the general population needs as well as the specific needs of people with disabilities is required to include people with disabilities in all pandemic response communications and activities. This includes providing public health communication, including information on COVID-19 prevention and government response measures in accessible formats. Identifying and removing barriers to prevention measures for COVID-19 may include measures such as providing additional support and equipment to carers of people with disabilities. Collecting and analysing disability-disaggregated data, and gathering lessons learned on what works in disability inclusion in COVID-19 responses will inform financial measures and economic planning. Finally, strengthening referral of people with disabilities to social protection schemes will facilitate food and other distribution from accessible locations.

## Conclusion

People with disabilities are a diverse group and are disproportionately impacted by COVID-19, both directly because of infection and indirectly because of restrictions to reduce the spread of the virus. Access to health care was limited in both rural and urban areas in Zimbabwe because of supply- and demand-side barriers. Seizing opportunities to prevent people with disabilities being further left behind and building on decades of progress on disability rights and economic empowerment may mitigate the widening of health inequalities in Zimbabwe.

## Appendix 1: Interview guide for people with disabilities

**Purpose:** To gain insights into people with disabilities’ access to health care services and their experiences when accessing or receiving care.

**Materials:**

- Notepad and pen/s
- Tape recorder

**Introduction:**

*Good morning/afternoon. My name is _____________. I am representing* ZAPP/PATAM/LC in conducting a research entitled: ***Building back better: Disability-inclusive health as a legacy of the COVID-19 pandemic in Zimbabwe***. You are invited to participate as a key informant as an individual with disability to share your experiences so that we can gain insights into people with disabilities’ access to health care services and their experiences when accessing or receiving care. This guide will be provided with the Information and Consent Sheet. Your responses will be treated with utmost confidentiality and your name and identity will remain anonymous. If you have questions about this research please feel free to ask any questions. You are free not to answer any questions you feel you can’t and also to stop the interview. Thank you in advance for your co-operation. The interview will take 30–60 min.

*Go through information sheet and ethics, confirm consent.*

**Background Information**

**Table:**

Interview Date and Time		
Interviewer		
Language of Interview		
Interview location (home, etc.)		
Town/State		
Gender	Male	Female
Age		
Marital Status		
Type of disability		
General Observations: (Anything which might impact how the interview is conducted, e.g. other present.)		

**A. About themselves:**

1. Please tell me about yourself (work, study, family, what is your routine generally like)
2. Please tell me about your family/household.

**Prompts:** Sources of income? Is there anyone else in the household who has major illness or disability? If so, who and what is their condition?

**B. About disability:**

**Now we are going to talk about your disability/impairment, and feel free again not to answer what you can’t answer and/or stop this interview.**

3. Please tell me about your impairment.

**Prompts:** Time of onset? If appropriate, ask what happened?

4. Do you need help to do the things you need to do every day? If so, do you receive any help or support from family or friends? What kind of support?

**Prompts:** Daily activities such as going to the bathroom, dressing, eating, going out – who helps or supports them and how? If yes, who and how? If not, why do you think that is?

**C. Health status and seeking services:**

5. How would you describe your health at this time?

**Prompts:** Do you have any health concerns? What is the main one that worries you? What are any co-morbidities you might have due to/caused by your impairment (if applicable)?

**D. Coronavirus**

*Now I would like to know more about your awareness of coronavirus.*

6. Have you heard of COVID-19 or coronavirus? [Describe/use terms used locally as needed. ***If no knowledge of COVID-19, skip to next section]***
7. How concerned are you about COVID-19/coronavirus?

**Prompts:** What are your concerns?

8. Do you think you are more at risk, less at risk or have the same risk as getting coronavirus or having serious illness from coronavirus compared to other people? Why?
9. What have you heard about ways you can protect yourself and others from getting coronavirus? What measures, if any, are you taking to protect yourself from coronavirus?

**Prompts***: (alter based on national policies/advice from local authorities):* Social distancing? Self-isolating? Frequent hand-washing? Wearing masks?

*Follow-up question/probe: For each measure mentioned but not done*: You mentioned [preventative measure] is a way to prevent getting coronavirus. What challenges, if any, do you face in following this? What, if anything, would help you to do [preventative measure]?

*Follow-up question/probe): For each measure followed*: Please tell me more about how you are doing this. What challenges, if any, do you face following this? What or who has helped you follow this?

10. Have you had coronavirus? If so, please describe your experience.

**E. Coronavirus and health care seeking**

*Now I would like to know more about your needs and activities, and whether these have changed since the new rules/arrangements because of COVID-19. So, for each, I would like you to think of a normal w eek before COVID-19, and then think about this week…*

11. Your impairment-related health needs (including rehabilitation, specialists etc)
• BEFORE

**Prompts:** What types of health services or products (e.g. medications, assistive devices such as a wheelchair, walking stick, hearing aids, etc.) do you use on a regular basis for your impairment? How do you typically access these?

If you do not use these services, why not? (e.g. don’t need them, cannot afford, don’t know how to access them).

- NOW

**Prompts:** Still able to access the same health services/products for your impairment?

■ *If no:* how have you been managing without these services/products? What challenges have prevented you from accessing these services/products?
■ *If yes:* what, if any, challenges have you faced accessing these services/products? What, if anything, has helped you maintain access to these services/products?

12. Any general health needs (including GP, pharmacy etc)
• BEFORE

**Prompts:** What types of health services or products (e.g. medications) do you use on a regular basis for your general health? How do you typically access these?

Thinking of a recent experience (pre-COVID), could you tell us step-by-step? How do you get to the clinic? At the clinic: how was physical accessibility, signage, experience of health care providers, price, equipment for their specific needs, denied care/treated differently from other patients? What worked well and what was difficult?

Have you ever been ill and didn’t access health services? Why? Focus on last two times.

- AFTER

**Prompt:** Still able to access the same health services/products for your general health?

*If no:* how have you been managing without these services/products? What challenges made it difficult for you to access these services/products?

*If yes:* what, if any, challenges have you faced accessing these services/products? What, if anything, has helped you maintain access to these services/products?

**F. Overall thoughts on access to health care services:**

13. Do you feel that your health care needs are met?

**Prompts**: What does having access to health care mean? Do you think that your health care needs are the same or different from people who don’t have your impairment/disability? Do you feel you receive the same or different quality of health care services as others? Do you feel you are treated same or differently?

14. Do you have any thoughts on what can make it easier for you to seek or access health care services (Examples: physical access, training of health care workers, treatment options, social interactions, education materials, financial support, etc.)?

**G. Other Information:**

Are there any other important issues which we haven’t covered which you would like to comment on or that you feel are important to addressing access to health care for people with disability?

**Thank you**

Thank you for taking the time to talk with me/us today. We have learned a great deal from you and your experiences. If you have any questions from our discussion please feel free to ask them.

## Appendix 2: Interview guide for key informants

**Introduction**

*Good morning/afternoon. My name is __________________. I am representing* ZAPP/PATAM/LC in conducting a research entitled: ***Building back better: Disability-inclusive health as a legacy of the COVID-19 pandemic in Zimbabwe***. You are invited to participate in as a key informant as an individual with disability to share your experiences on disability to build the evidence base that characterises the impacts of the coronavirus pandemic amongst people with disabilities. This guide will be provided with the Information and Consent Sheet. Your responses will be treated with utmost confidentiality and your name and identity will remain anonymous. If you have questions about this research please feel free to ask any questions. You are free not to answer any questions you feel you can’t. Thank you in advance for your co-operation. The interview will take 30–60 min.

**Table:**

*Code number*	
*Interview date and time*	
*Interview venue and location*	
*Interviewer*	
*Interviewee*	
*Job title*	
*Organisation*	

**Section 1: Key informant background**

I’m now going to ask you some questions about your background.

1. Please tell me more about your role as [job title].

**Prompt:** What activities do you do in this role?

2. How, if at all, has your work been affected by COVID-19?

**Prompt:** Changes in types or way of doing activities?

3. Is your organisation involved in COVID-19 response (either direct – e.g. prevention, treatment; or indirect – e.g. economic responses)?
  - *If yes*: In what ways? Do you think people with disabilities are adequately included? Why/why not?

**Section 2: COVID-19 impact and responses**

I’m now going to ask you some questions about the impact of COVID-19 on different areas of daily life that people in your area may have experienced. ***[Note to interviewers: start by asking about all people and then focus in on people with disabilities]***

4. What challenges, if any, have people faced in following COVID-19 prevention measures (e.g. social distancing, staying at home, handwashing/hygiene practices)?
  - Are the challenges the same or different for people with disabilities? If different, explain in what ways?
5. What do you think has been the impact of COVID-19 on…
  [*Note to* interviewers: focus on key informant’s area of expertise. For each, explore how these do or do not differ compared to people without disabilities. Clarify if these were existing challenges or new/made worse due to COVID-19]
  a. Work (and other livelihood activities)?
  • Is this impact the same or different for people with disabilities? If different, in what ways?
  • [If an impact for people with or without disabilities]: What, if any, policies/programmes /solutions are being implemented to address this?
    ■ *If yes*: What are the strengths/weaknesses of this programme? Are these strengths/weaknesses the same for people with disabilities compared to people without disabilities? Why/why not?
      ◦ Do you think this programme/policy/activity meets the needs of people with disabilities? Why/why not? (*Probes*: type of service adequate? Method of delivery? How people access it?)
    ■ *If none*: What do you think would be helpful in addressing this? How, if at all, would this need to be adapted to include people with disabilities? *Probes*: type of service? Way service delivered?
  b. School?
  • Is this impact the same or different for people with disabilities? If different, in what ways?
  • [If an impact for people with or without disabilities]: What, if any, policies/programmes/solutions are being implemented to address this?
    ■ *If yes*: What are the strengths/weaknesses of this programme? Are these strengths/weaknesses the same for people with disabilities compared to people without disabilities? Why/why not?
      ◦ Do you think this programme/policy/activity meets the needs of people with disabilities? Why/why not? (*Probes*: type of service adequate? Method of delivery? How people access it?)
    ■ *If none*: What do you think would be helpful in addressing this? How, if at all, would this need to be adapted to include people with disabilities? *Probes*: type of service? Way service delivered?
  c. Accessing health care (e.g. doctors, hospitals, pharmacy)?
  • Is this impact the same or different for people with disabilities? If different, in what ways?
  • [If an impact for people with or without disabilities]: What, if any, policies/programmes/solutions are being implemented to address this?
    ■ *If yes*: What are the strengths/weaknesses of this programme? Are these strengths/weaknesses the same for people with disabilities compared to people without disabilities? Why/why not?
      ◦ Do you think this programme/policy/activity meets the needs of people with disabilities? Why/why not? (*Probes*: type of service adequate? Method of delivery? How people access it?)
    ■ *If none*: What do you think would be helpful in addressing this? How, if at all, would this need to be adapted to include people with disabilities? *Probes*: type of service? Way service delivered?
  d. Ability to get food and other essentials?
  • Is this impact the same or different for people with disabilities? If different, in what ways?
  • [If an impact for people with or without disabilities]: What, if any, policies/programmes/solutions are being implemented to address this?
    ■ *If yes*: What are the strengths/weaknesses of this programme? Are these strengths/weaknesses the same for people with disabilities compared to people without disabilities? Why/why not?
      ◦ Do you think this programme/policy/activity meets the needs of people with disabilities? Why/why not? (*Probes*: type of service adequate? Method of delivery? How people access it?)
    ■ *If none*: What do you think would be helpful in addressing this? How, if at all, would this need to be adapted to include people with disabilities? *Probes*: type of service? Way service delivered?
  e. Social care needs (e.g. personal assistance, social protection)
  • Is this impact the same or different for people with disabilities? If different, in what ways?
  • [If an impact for people with or without disabilities]: What, if any, policies/programmes/solutions are being implemented to address this?
    ■ *If yes*: What are the strengths/weaknesses of this programme? Are these strengths/weaknesses the same for people with disabilities compared to people without disabilities? Why/why not?
      ◦ Do you think this programme/policy/activity meets the needs of people with disabilities? Why/why not? (*Probes*: type of service adequate? Method of delivery? How people access it?)
    ■ *If none*: What do you think would be helpful in addressing this? How, if at all, would this need to be adapted to include people with disabilities? *Probes*: type of service? Way service delivered?
  f. Impairment-specific health care (e.g. rehabilitation, medications, psychiatry)
  • What, if anything, has been the impact?
  • [If an impact] What, if any, policies/programmes/solutions are being implemented to address this?
    ■ *If yes*: What are the strengths/weaknesses of this programme?
      ◦ Do you think this programme/policy/activity meets the needs of people with disabilities? Why/why not? (*Probes*: type of service adequate? Method of delivery? How people access it?)
    ■ *If none*: What do you think would be helpful in addressing this?
  g. Impact on any other areas?

**Section 3: Wider context**

6. Do you feel that the needs of people with disabilities have been adequately considered by the government/programme implementers during the COVID-19 epidemic? Why/why not?
7. Is there anything else you would like to say about the impact of the coronavirus epidemic on people with disabilities?
